# Increased Expression of Toll-Like Receptors 4, 5, and 9 in Small Bowel Mucosa from Patients with Irritable Bowel Syndrome

**DOI:** 10.1155/2017/9624702

**Published:** 2017-01-29

**Authors:** Aldona Dlugosz, Katherina Zakikhany, Nathalie Acevedo, Mauro D'Amato, Greger Lindberg

**Affiliations:** ^1^Department of Medicine, Karolinska Institutet and Center for Digestive Diseases, Karolinska University Hospital, Huddinge, Stockholm, Sweden; ^2^Unit for Laboratory Surveillance of Viral Pathogens and Vaccine Preventable Diseases, Public Health Agency of Sweden, Solna, Sweden; ^3^Department of Clinical Science and Education, Karolinska Institutet, Stockholm, Sweden; ^4^Sachs' Children and Youth Hospital, Södersjukhuset, Stockholm, Sweden; ^5^Unit of Clinical Epidemiology, Department of Medicine Solna, Karolinska Institutet, Stockholm, Sweden; ^6^BioDonostia Health Research Institute and Ikerbasque (Basque Science Foundation), Bilbao, Spain

## Abstract

The aim of our study was to compare patients with irritable bowel syndrome (IBS) and healthy controls regarding the expression of toll-like receptors 2, 4, 5, and 9 (TLR2, TLR4, TLR5, and TLR9), the primary mucosal receptors of bacterial components, in small and large bowel mucosa.* Methods.* We analysed biopsies from jejunum and sigmoid colon of 22 patients (17 females) with IBS aged 18–66 (median: 39) years and 14 healthy volunteers (12 females) aged 22–61 (median: 42) years. Eight patients had constipation-predominant IBS (C-IBS), 7 had diarrhoea-predominant IBS (D-IBS), and 7 had IBS without predominance of constipation or diarrhoea. We analysed mRNA levels for TLRs using quantitative PCR and distribution of TLRs in mucosa using immunohistochemistry.* Results.* We found increased mRNA expression of TLR4 (mean fold change 1.85 ± 0.31 versus 1.0 ± 0.20; *p* < 0.05), TLR5 (1.96 ± 0.36 versus 1.0 ± 0.20; *p* < 0.05) and TLR9 (2.00 ± 0.24 versus 1.0 ± 0.25; *p* < 0.01) but not of TLR2 in the small bowel mucosa from patients with IBS compared to the controls. There was no significant difference in mRNA levels for TLRs in colon mucosa between patients and controls.* Conclusion.* Upregulation of TLR4, TLR5, and TLR9 suggests the involvement of bacteria or dysregulation of the immune response to commensal flora in small bowel mucosa in IBS patients.

## 1. Introduction

Irritable bowel syndrome (IBS) is one of the most common functional gastrointestinal disorders and among the most frequent causes of gastroenterology consultations in the western world [1]. IBS is characterised by the presence of abdominal pain or discomfort that is associated with alterations in bowel habits. The pathogenesis of IBS is unclear but a role for low-grade inflammation and immunological alterations in the development of IBS has become evident through a number of previous studies [2, 3].

Intestinal epithelial surfaces are constantly exposed to antigens from the normal microbiota, food antigens, and pathogenic microorganisms. Bacterial wall products play an important role in activation of immune and nonimmune cells of the intestinal mucosa. The intestine needs to be able to respond to pathogenic organisms while at the same time maintain tolerance to the commensal flora. The abilities of epithelial cells to detect bacterial cellular components require the expression of pattern recognition receptors (PRRs) that recognize repetitive patterns present on Gram-positive and Gram-negative bacteria, fungi, viruses, and parasites. Toll-like receptors (TLR) belong to the family of PRRs and are present in macrophages of the lamina propria, dendritic cells, Paneth cells, and intestinal epithelial cells [4]. So far, 10 TLRs have been reported in humans [5]. Among them TLR2 is regarded as the PRR for Gram-positive bacteria, through its recognition of bacterial lipoproteins and peptidoglycans; TLR4 recognizes lipopolysaccharide (LPS) from Gram-negative bacteria, TLR5 is PPR for flagellin, a protein from motile Gram-positive and Gram-negative bacteria, and TLR9 mediates the cellular response to bacterial DNA [5, 6]. After recognition of their ligands TLRs activate intracellular signalling pathways leading to production of proinflammatory cytokines and chemokines [7]. The continuous presence of bacteria in the immediate proximity of mucosal PRRs indicates that PRR signals are under precise control to avoid overstimulation. The TLR signalling pathway can be regulated at the receptor level, as well as at several sites along the signalling cascade [8]. It is possible that there may be crosstalk between various TLR family members in the maintenance of intestinal inflammation, and the balance between intestinal homeostasis versus intestinal injury may be a reflection of the relative balance between TLRs and their associated signalling molecules. Alternatively, different TLRs within the intestine may be more or less susceptible to upregulation by different physiological stressors [9]. Previous studies detected mRNA expression of* TLR2*,* TLR4*,* TLR5,* and* TLR9* in both large and small intestinal epithelial cells (IECs) [10], although TLR expression and signalling appear to be downregulated in physiological conditions [11]. The role of TLRs in normal homeostatic processes and in the pathogenesis of chronic inflammatory diseases has become apparent [12].

Previous studies have found an increased expression of TLR2 and TLR4 on both mRNA and protein levels in inflammatory bowel disease [13–15] and coeliac disease [16].* TLR4 *expression is also increased in experimental and human necrotizing enterocolitis [17]. Surprisingly, expressions of the* TLR5* gene and its corresponding protein seem to be decreased in the mucosa of patients with ulcerative colitis [18]. There are a few data about the role of TLRs in IBS. Schoepfer et al. [19] described the immunoreactivity to bacterial flagellin among IBS patients. Brint et al. [20] described increased expression (mRNA and protein) of TLR4 and TLR5 in colon biopsies from patients with IBS. TLR9 rs5743836 (A/g) gene polymorphism is possibly associated with the phenotype of IBS-D [21] but so far the role of TLRs in the pathogenesis of IBS has not been explored. The presence of low-grade inflammation and immunological alterations in IBS patients suggests a possible involvement of TLRs in the development of IBS.

The aim of our study was to compare patients with IBS and healthy controls regarding the mRNA and protein expression of TLR2, TLR4, TLR5, and TLR9, which are the primary mucosal sensors of bacterial patterns, in small and large bowel mucosa.

## 2. Materials and Methods 

### 2.1. Patients

Patients who fulfilled Rome-II criteria for IBS [22] were prospectively recruited from Karolinska University Hospital, Huddinge Outpatient Clinic, in the time period from January 2006 to December 2011. The Rome-III criteria were published in April 2006 but a validated Swedish translation of the Rome-III modular questionnaire became available first in 2009. We therefore stayed with the Rome-II criteria throughout this study. Exclusion criteria were pregnancy, severe concomitant diseases, gastrointestinal comorbidities, current infection, and the lack of informed consent. A total of 22 patients (17 females and 5 males) with a median age of 39 (range 18–66) years and a median duration of IBS symptoms of 6.5 years (range 0.6–33.2 years) were investigated. Diarrhoea-predominant IBS (D-IBS) was present in 7 patients (32%), 8 patients (36%) had constipation-predominant IBS (C-IBS), and 7 patients (32%) had IBS without predominance of constipation or diarrhoea.

### 2.2. Controls

The control group comprised 14 healthy volunteers (12 females) in whom IBS and all other functional bowel disorders had been excluded by medical interview and a validated questionnaire for the Rome-II symptom criteria. The median age of the controls was 42 (range 22–61) years. Neither patients nor controls had been treated with antibiotics or probiotics for one month prior to biopsy taking.

### 2.3. Mucosa Biopsies

Mucosa specimens from the proximal jejunum were taken with a Watson capsule in 22 patients and 14 controls. The capsule was swallowed by the subject and brought by peristalsis to a position distal to the ligament of Treitz as determined by fluoroscopy. The same day all patients and controls underwent sigmoidoscopy and mucosa specimens from the distal sigmoid colon were taken using biopsy forceps. We did not use any cleansing of the colon before the examination.

### 2.4. RNA Extraction and cDNA Synthesis

Jejunum and colon biopsies to RNA extraction were immediately stabilized in 500 *µ*l RNA later solution (Qiagen, Germany) according to the manufacturer's instructions and stored at −80°C until further use. Prior to RNA extraction, biopsy samples were homogenized with a pestle in 1.5 ml Eppendorf tubes containing 500 *µ*l RLT lysis buffer. Total RNA was extracted using the RNeasy Mini RNA Extraction Kit (Qiagen, Germany) including an on-column DNase treatment procedure, following the manufacturer's instructions. Extracted RNA was quantified with a NanoDrop spectrophotometer (Thermo Fisher Scientific) and stored at −80°C until further usage. A standard Taq-PCR was performed to detect traces of DNA. Further DNA contaminations were removed using the RQ1 RNase-Free DNase system (Promega). First-strand cDNA was prepared using the RNA-to-cDNA Kit (Applied Biosystems) according to the manufacturer's instructions. cDNA was prepared in a total volume of 20 *µ*l containing 1x RNA-to-cDNA Master Mix and 500 ng of total RNA and nuclease-free water (Promega). cDNA samples were stored at −20°C until further usage.

### 2.5. SYBR GREEN Quantitative PCR

Relative transcript abundance of selected target genes was determined with 2^−ΔΔCt^ method [23, 24] using the 7500 SDS Software v1.4.1 (Applied Biosystems). Each reaction was run in a final volume of 25 *μ*l containing the following reagents: 12.5 *μ*l of Power SYBR GREEN PCR reaction mix, 3 *μ*l of 1 *μ*M forward primer, 3 *μ*l of 1 *μ*M reverse primer, 1 *µ*l cDNA (1 : 10 diluted in water), and 5.5 *µ*l water (Table 1). The PCR profile was 1 cycle at 50°C for 2 min; 1 cycle at 95°C for 10 min; 45 cycles at 95°C for 15 s; and 60°C for 60 s, with fluorescence acquisition at 60°C. hGAPDH was used for internal normalization and relative expression values were assessed from triplicates per real-time PCR assay relative to a calibrator value (healthy controls).

### 2.6. Immunohistochemistry

Immunohistochemistry was performed on paraffin embedded sections in a subset of patients (*n* = 10) and controls (*n* = 10) using goat polyclonal antibodies to TLR2 and TLR5 as primary antibodies and rabbit polyclonal anti-goat as a secondary antibody. TLR4 expression was analysed using rabbit polyclonal antibody as a primary antibody and goat anti-rabbit as a secondary antibody. For TLR9 expression we used mouse monoclonal antibody as primary antibody and goat anti-mouse as a secondary antibody (all from Abcam, Cambridge, UK). Development of the signal was achieved by an immunoenzymatic assay with streptavidin-biotin complex (Dako, Glostrup, Denmark). Control experiments were performed omitting the primary antibody. In order to enhance detection of low levels of TLR4 protein in intestinal mucosa we used Tyramide Signal Amplification (TSA) Kit (Invitrogen, Carlsbad, CA, USA) according to the method described by Ungaro et al. [25].

### 2.7. Statistical Analysis

Statistical analysis was done using Student's *t*-test for fold change values. Fold changes were expressed as means and standard error relative to controls. Significance level was set at *p* < 0.05.

### 2.8. Ethical Considerations

All parts of the study were approved by the Regional Ethical Review Board in Stockholm and followed the ethical guidelines of the Helsinki Declaration. Informed consent was obtained from all patients and controls at the time of biopsy sampling.

## 3. Results

### 3.1. The mRNA Expression of Toll-Like Receptors

**Figure 1:**
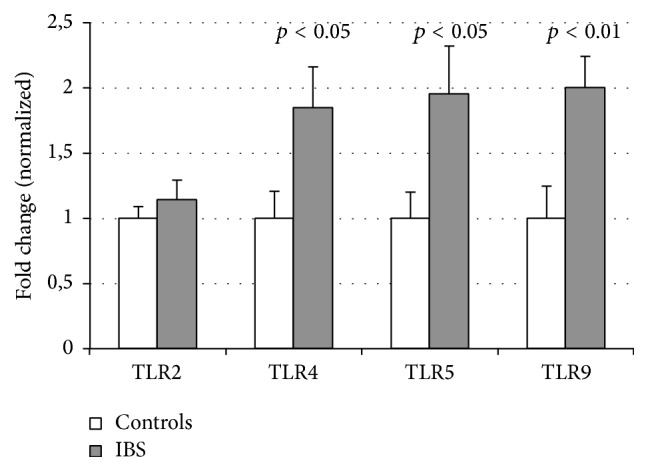
The relative mRNA expression of TLRs in small bowel of patients with IBS and controls. Relative mRNA levels are expressed as a normalized fold change (1 for healthy controls).

The mRNA expression in jejunum mucosa of* TLR4 *(mean fold change 1.85 ± 0.31 versus 1.0 ± 0.20; *p* < 0.05), TLR5 (1.96 ± 0.36 versus 1.0 ± 0.20; *p* < 0.05), and* TLR9* (2.00 ± 0.24 versus 1.0 ± 0.25; *p* < 0.01) was higher among patients with IBS compared to controls (Figure 1). No difference between patients and controls was found in the expression of* TLR2* in jejunum.

**Figure 2:**
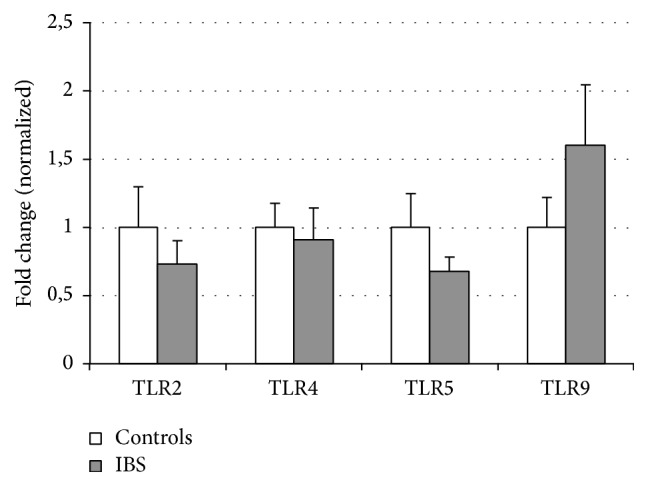
The relative mRNA expression of TLRs in large bowel of patients with IBS and controls. Relative mRNA levels are expressed as a normalized fold change (1 for healthy controls).

Similarly, the expressions of* TLR2*,* TLR4*,* TLR5,* and* TLR9* were measured in biopsies from the sigmoid colon in the same patients and controls. However, we found no significant difference between IBS patients and controls in the expression of these receptors in colon (Figure 2).

We found differences in* TLRs* expression between jejunum and colon in healthy subjects. Analysis of uncorrected (raw) expression data revealed that median* TLR2* expression was higher in the jejunum (13.3 IQR (7.6–16.1)) than in the colon (7.1 (5.5–10.5)). In contrast, the median expressions of* TLR4* (2.0 (1.4–5.5)),* TLR5* (4.4 (3.2–9.3)), and* TLR9* (2.8 (1.4–7.7)) in the jejunum were lower than in the colon (9.1 (7.7–15.2); 20.4 (13.5–31.2); and 12 (5.3–16.1), resp.).

### 3.2. Immunohistochemical Expression of Toll-Like Receptors

#### 3.2.1. TLR2

We found weak immunostaining in small and large bowel epithelium in samples obtained from healthy controls. Stronger positivity in both* lamina propria* and epithelium appeared in small and especially large bowel samples (crypts and surface epithelium) obtained from IBS patients.

#### 3.2.2. TLR4

**Figure 3:**
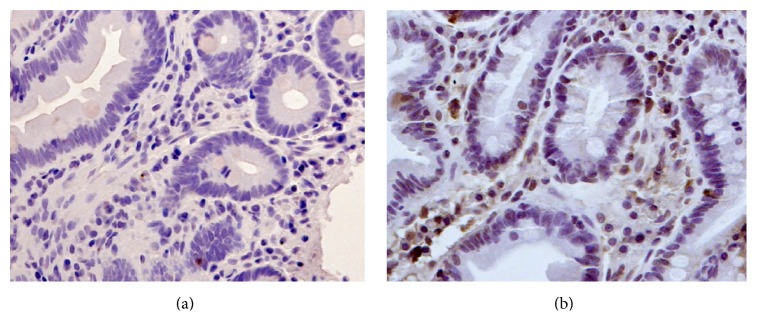
Immunohistochemistry images of TLR4 staining, original magnification ×20. (a) Biopsy from jejunum of a healthy control. (b) Biopsy from jejunum of a patient with IBS showing positive staining mainly in migrating cells of* lamina propria*.

Both small and large bowel samples taken from healthy controls were negative for TLR4 in immunostaining. In patients with IBS we found positive staining mainly in cytoplasm of migrating cells in the* lamina propria* in both small and large bowel (Figure 3).

#### 3.2.3. TLR5

IBS patients showed increased expression of TLR5 mostly in* lamina propria* but also in the striated border of villous epithelial cells. Healthy controls showed positivity only in the epithelium.

#### 3.2.4. TLR9

**Figure 4:**
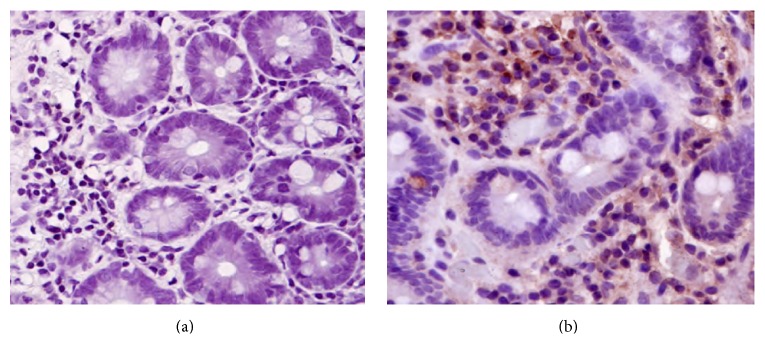
Immunohistochemistry images of TLR9 staining, original magnification ×20. (a) Biopsy from jejunum of a healthy control. (b) Biopsy from jejunum of a patient with IBS showing positive staining mainly in the cytoplasm of migrating cells of* lamina propria*.

TLR9 was not detected in small bowel of healthy individuals but was detected in epithelium and* lamina propria* in colon samples. IBS patients had upregulated TLR9 expression in cytoplasm of migrating cells in the* lamina propria* in the small bowel and in both epithelium (surface epithelium and crypts) and* lamina propria* in the large bowel (Figure 4).

## 4. Discussion

Although the molecular alterations leading to IBS remain elusive, there is growing evidence that an altered regulation of immune activation and low-grade inflammation are involved in the pathogenesis of IBS [26]. We found significantly upregulated expression of TLR4, TLR5, and TLR9 in small bowel mucosa of patients with IBS. To our knowledge, our study is the first to assess TLR expression in both small bowel and large bowel of patients with IBS and healthy controls. Intestinal epithelial cells (IECs) in the human colon have been thought to express most of the TLRs. However, in vitro data have shown that TLR expression and signalling in IEC appear to be downregulated in healthy individuals [11, 27]. In contrast, upregulation of TLR expression in IEC has been seen in inflammatory bowel diseases of the colon [12]. We did not find any previous studies describing TLR expression in the human jejunum.

Cario and Podolsky [13] analysed TLR2, TLR3, TLR4, and TLR5 protein levels in colonic biopsies and found that TLR4 protein levels were higher in both the inflamed and the noninflamed colonic mucosa of patients with inflammatory bowel disease compared to controls. McKernan et al. [28] found that TLR4 was increased in the colon of rats using two different animal models of IBS. Brint et al. [20] analysed the genetic expression of TLRs in colon mucosa from patients with IBS and found upregulation of* TLR4* and* TLR5* in colon.

Our observation that* TLR4* expression is increased in IBS patients is consistent with published data showing that TLR4 is upregulated in the intestine in association with increased inflammation [13]. However, we found increased transcript expression of TLR4 in small bowel mucosa but not in the large bowel. In our study, although we found differences in staining for TLRs between IBS patients and healthy controls, we did not confirm the upregulated expression of TLRs in large bowel. Finding* TLR4, TLR5,* and* TLR9* upregulation in small bowel suggests that disturbances in the small bowel can be more important than those in the large bowel for developing of IBS. We found differences in* TLRs* expression between small and large bowel in healthy subjects. Median* TLR2* expression was higher in the jejunum than in the colon. However, the median expressions of* TLR4, TLR5*, and* TLR9* were lower in the jejunum than in the colon. Observed differences in healthy subjects can be due to different microbiota compositions of the small and large bowel.

It is not clear why TLR4, TLR5, and TLR9 would be increased in the small intestine of patients with IBS. Do bacterial products passing through an excessively permeable epithelial barrier overstimulate these receptors?

TLR4 recognizes LPS contained in the cell wall of Gram-negative bacteria, TLR5 recognizes flagellin, and TLR9 recognizes nonmethylated CpG motifs abundantly contained in bacterial DNA. The increased expression of TLR4, TLR5, and TLR9 that we detected in patients with IBS is possibly in response to an increase in their endogenous ligands by disruption of the intestinal barrier and increased intestinal permeability, two suggested pathogenic mechanisms in IBS [29]. Indeed, previous studies reported upregulation of these receptors in patients with inflammatory bowel disease (IBD) in which disruption of epithelial barrier and bacterial translocation do occur.

The other question is if TLRs react to the presence of pathogenic bacteria or rather pathologically react to commensal flora. TLR9 is involved in barrier function and TLR9 signalling mediates, at least in part, the anti-inflammatory effects of natural commensal-origin DNA on the gut [6]. In colon, epithelial TLR9 actively contributes to intestinal homeostasis [30]. Alterations in microflora, described in IBS patients, may affect the ability of the microbiota to adhere to the mucosa and influence innate immunity activation [31]. It remains to be elucidated whether the increased TLR expression demonstrated in this study is associated with mucosal immune activation. It is known that TLRs elicit intracellular signalling pathways in target cells, which result in the activation of gene expression and synthesis of a broad range of molecules, including cytokines, chemokines, cell adhesion molecules, immunoreceptors, and factors involved in antimicrobial responses. Increased levels of proinflammatory cytokines and small and large bowel infiltration with variety of immune cells, including macrophages, mast cells, T cells, and B cells, have been previously reported in IBS patients [32].

To better clarify the cellular elements expressing TLR in the intestine we decided to analyse TLR expression with both RT-PCR and immunohistochemistry. Since expression of TLRs (particularly TLR4) is normally very low, assaying for changes in its expression is sometimes difficult because of the limitations of detection methods. Although polymerase chain reaction (PCR) based method has an advantage to detect such fine biological changes, results of intestinal expression of TLRs by PCR method usually vary within the whole mucosa. Both IEC and infiltrating immune cells potentially express TLR4 making the expression level vary according to how many TLRs expressing cells exist in each sample. IHC may be a good tool to overcome this problem since we may look at expression of TLRs in various cell types in a larger tissue sample. To overcome the problem with low expression of TLR4 we used TSA for IHC [25]. In IHC IBS cases seemed to express more TLR4 than healthy controls in colon but this impression may be caused by TSA effect inflating a small difference.

In order to understand the discrepancies between our findings and those of Brint et al. [20] regarding TLR4 and TLR5 expression in large bowel we think it is important to analyse studied populations. Although the numbers of patients and controls in the two studies were similar, the study by Brint et al. comprised women only (in our study 23% men). With regard to IBS subtypes, the ratio of alternators : diarrhoea-predominant : constipation-predominant was 14 : 7 : 5 in the study by Brint et al. and 7 : 7 : 8 in ours. This means that the proportion of patients with diarrhoea was 1.5 times higher in the study by Brint et al. compared to ours. We cannot exclude that in the colon the higher expression of TLRs is driven by a difference between patients with diarrhoea and controls that can explain obtained results. On the other hand Belmonte et al. [33] reported upregulation of TLR2 and TLR4 expression in colonic mucosa in patients with mixed subtype of IBS but not in those with constipation or diarrhoea-predominant IBS. Upregulation was localized to the epithelial cells. The study analysed 26 patients (respective ratio 7 : 10 : 9). Differences in TLRs expression in colon may be due to small sample sizes and the heterogeneity of the IBS population.

Our study has several limitations. One of them is the small sample size that did not allow us to compare TLRs expressions between different subgroups. Further studies are needed to dissect which alterations in TLRs expression are related to the different IBS subgroups as suggested by the results of Brint et al. [20] and Belmonte et al. [33].

In summary, our study is the first that analyses the expression of TLRs at the transcriptional and protein level in small bowel of patients with IBS, with the strength of including samples from both small and large intestine, from the same individuals at the same time-point. Our results suggest that altered expression of TLRs in the small bowel is involved in the pathogenesis of IBS.

## Figures and Tables

**Table 1:** Primers used for TLR expression analysis. FFW, forward primer, REV, reverse primer.

TLR2	FFW:	TGGGCAGTCTTGAACATTT
	REV:	GAATCTTAGTGAAGGTGTCCAT
TLR4	FFW:	TTCAAGGTCTGGCTGGTT
	REV:	CGAGGTAGTAGTCTAAGTATGCTA
TLR5	FFW:	GTTCCTGACACTACTACAAGATTC
	REV:	GCTGGAGCAGATGAGAGT
TLR9	FFW:	CACCAGCCTTTCCTTGTC
	REV:	TGGCAGAGTCTAGCATCA
GAPDH	FFW:	GGTCGGAGTCAACGGATT
	REV:	ATCGCCCCACTTGATTTTG

## Abbreviations

C-IBS: Constipation-predominant irritable bowel syndrome
CD: Crohn's disease
D-IBS: Diarrhoea-predominant irritable bowel syndrome
DNA: Deoxyribonucleic acid
hGAPDH: Human glyceraldehyde 3-phosphate dehydrogenase
IBD: Inflammatory bowel disease
IEC: Intestinal epithelial cell
IBS: Irritable bowel syndrome
LPS: Lipopolysaccharide
mRNA: Messenger ribonucleic acid
PRR: Pattern recognition receptor
PCR: Polymerase chain reaction
TLR: Toll-like receptor
TSA: Tyramide signal amplification.
